# Report on the Changes in the Composition and Properties of the Milk in the Human Female, Produced by Menstruation and Pregnancy; Also, on the Food Most Proper for Infants When Deprived of the Milk of the Mother

**Published:** 1860-10

**Authors:** N. S. Davis

**Affiliations:** Chicago, Ill.


### THE CHICAGO MEDICAL EXAMINER

VOL. I. OCTOBER, 1860. No. 10

### ORIGINAL COMMUNICATIONS.

### REPORT

*On the Changes in he Composition and Properties of the Milk in the Human Female, produced by Menstruation and Pregnancy; also, on the Food most Proper for Infants when deprived of the Milk of the Mother.*

**Presented to the Illinois State Medical Society, May, 1860.**

BY N. S. DAVIS, M. D., &C., CHICAGO, ILL.

At the annual meeting of the American Medical Association, held in May, 1856, I had the honor to present a brief report on the chemical and microscopic changes that take place in the milk of the human female during menstruation and pregnancy.

The Association in accepting that report requested a continuance of the investigation, and by a subsequent resolution, imposed upon me the additional duty of reporting on the food most proper for infants when deprived of the milk of the mother. In continuance of the investigation commenced in the former report, observations and notes have been made concerning twelve additional cases of pregnancy and eight of menstruation. In six of these cases, four of pregnancy and two of menstruation, the milk was obtained for examination and analysis in the same manner as detailed in the former report.

The examinations with the Micerscope revealed nothing that was not fully described and represented in that report.

In all the cases of pregnancy there was a noticeable change in the quantity of milk globules; the full sized globules being fewer, while the mere granules were increased in number. In the milk obtained while the mother was menstruating the change was much less observable. The observations and analyses contained in the former report led us to the following conclusions, viz.:

1st. The occurrence of pregnancy, during lactation, produces a very marked diminution of all the solid or nutritive constituents of the milk.

2nd. In examining the separate proximate elements, it will be observed that a much greater relative diminution takes place in the caseine, the butter or oil, and the salts, than in the sugar and extractive matter.

Having now completed a direct comparison between the microscopic appearances and chemical composition of the milk of six different mothers while pregnant, with specimens of milk from the same mothers when in the third or fourth month of lactation without pregnancy, but preserving in each case all the circumstances connected with diet and exercise as nearly uniform as possible, I think the conclusions just stated clearly demonstrated. It would have been easy to have multiplied the number of analyses, had it been proper to compare the composition of the milk of one female in a pregnant state with that of another not pregnant. But the well known fact that milk from different individuals, differs much in the relative proportion of its constituents, would render any conclusions drawn from such a comparison more or less unsatisfactory. Hence I have confined myself in this, as in the former report, to such cases only as would permit a comparison of the two conditions in the same individual.

*Changes in the Qualities of the Milk.*—The properties of milk doubtless depend partly on the relative proportion of its constituents, partly on the more or less perfect elaboration of these, and in part also upon the admixture of accidental or foreign ingredients

Careful examinations were made relative to the health of both mothers and children, which have been noted since the previous report. In eight, the mothers continued to enjoy as good health as is usual during the first three months of pregnancy, when not complicated with lactation; but their children all began to show signs of insufficient nutrition, coupled with more or less disturbance of the stomach and bowels, before the completion of the second month of pregnancy. In two the children continued well nourished and healthy, but the mothers both became rapidly anemic, and the organic nervous system extremely excitable, as indicated by palpitations of the heart, muscular weakness, and inability to endure even moderate exercise.

In the remaining two, both mothers and children continued to enjoy a fair degree of health until the fourth month of pregnancy, but the children drank freely of cow's milk and sometimes took other nourishment, and the quantity of milk furnished by the mothers was small.

Of the eight cases of menstruation during lactation, in three both mothers and children continued well; in three others the mothers continued well, but their children were unusually fretful, and subject to frequent turns of slight diarrhœa; in the remaining two the mothers became affected with erythematic inflammation of the mouth, profuse leucorrhœal discharges after each menstrual period, and much general debility, while the children showed no other signs of ill health than unusual peevishness.

From all the foregoing facts and analyses, I am led to infer that the occurrence of pregnancy during the ordinary period of lactation either speedily reduces the quantity of milk secreted, or lessens the proportion of solid or nutritive constituents, to such a degree as to render it insufficient for the proper nourishment of a child over six months old. In a small proportion of cases, however, the milk secreted continues abundant and of good quality, but the health of the mother rapidly declines; while in a still smaller proportion of cases, the mother and child both continue well nourished and healthy. Taking these statements as correct, they suggest two questions of much importance.

First: Whenever pregnancy occurs during lactation and the mother or child, or both, are found to exhibit symptoms of anemia, should the child be immediately removed from the breast; or should an effort be made to devise such diet and medicine as would enable the mother to assimilate sufficient for herself, and both the intra and extra uterine off-spring?

Second: If an attempt is made to sustain the mother and permit lactation to continue, what dietetic and therapeutical measures would be most efficient for that purpose?

The defects in the milk, as shown by analysis, as well as the condition of the mother, readily suggest a diet containing abundance of caseine, gluten, or albumen, and such tonics as the compound syrup of phosphates, or still better, the hypophosphites of soda, potassa, lime, and iron; but your committee cannot report a sufficient number of clinical cases to determine the actual value of such a course. On the contrary, nothing short of an early removal of the child from the breast has been found safe in the great majority of cases thus far observed. And this leads directly to the consideration of the second question propounded to me by the Association, namely, "what food is most suitable for infants when from any cause it becomes necessary to deprive them of the mother's milk at too early a period?" Very few questions could be stated having a more direct bearing upon the preservation of life and health, than this. The ratio of mortality during the first five years of life is not less than one per cent. of the population in cities. And if this inquiry was restricted to such infants as are deprived of the mother's milk during the first six months after birth, it is quite certain that the ratio would be doubled. Dr. Reese, in his report on infant mortality made to the American Medical Association, very justly places impure milk and improper food, used during the first year of infancy, among the prominent causes of such mortality. Hence the subject is one of sufficient importance to be worthy of the most careful investigation. There are two methods by which inquiries may be conducted, on this subject, viz.: the rational and the empirical. The first embraces a careful consideration of the following questions:—

1st. What proximate and elementary constituents must food contain to render it capable of affording perfect nutrition to the infant?

2nd. What articles of food contain such constituents? and what state, in relation to fluidity, temperature, &c., is best adapted to the condition of the digestive apparatus in infancy?

The second method of inquiry consists in the direct administration of different articles of food to a sufficient number of children, and for a sufficient length of time to determine their absolute and relative value.

To do this satisfactorily would almost necessarily require the control of a Foundling Hospital or Orphan Asylum in which a considerable number of infants were admitted. Without any such facilities for pursuing the empirical method of inquiry, we must be content with the simple announcement, derived from the general experience of the profession, that infants are capable of being fully nourished and all their tissues brought to a healthy degree of developement on a considerable variety of alimentary substances. The most important of these are, the milk of animals; panada or pap prepared from wheaten bread or biscuit; oatmeal or groats; and the several farinaceous articles such as corn starch, arrow-root, rice, &c.

If we subject these several articles to the rational method of inquiry, as already defined, we shall find each of them containing all the proximate and inorganic constituents necessary for nourishing the various structures of the human body, but in very variable proportions. Thus if we assume that all substances, capable of sustaining perfect nutrition in infants, must contain more or less nitrogenous, carbonaceous, and saline or inorganic matter, and subject those already named to an analytical comparison, we shall find in the cow's milk the three classes of alimentary principles in nearly the same aggregate proportion as in healthy milk from the human female; in the preparations derived from wheat and oats the nitrogenous elements represented by gluten or vegetable albumen though less than in milk are abundantly sufficient; while in those derived from rice, arrow-root, &c., the nitrogenous constituents are very deficient in quantity, and the carbonaceous, as represented by starch and sugar, relatively in excess. This will be more forcibly illustrated by a glance at the proximate analysis of these several substances. Thus, if we add to wheat flour, oatmeal, and rice flour respectively, a sufficient quantity of water to render them capable of being compared with cow's milk, we shall find them to contain, in 1000 parts,

| | Nitrogenous El. | Carbonaceous. | Saline. | Water. |
|---|---|---|---|---|
| Cow's milk,.................. | 44,80 | 79,00 | 6,00 | 870,00 |
| Wheat flour, (diluted with water),. | 15,74 | 110,65 | 3,46 | 870,00 |
| Oatmeal, do. do. . | 13,25 | 101,79 | 5,88 | 870,00 |
| Rice flour, do. do. . | 5,08 | 193,62 | 0,70 | 870,00 |

The obvious deductions from this table are in strict conformity with the teachings of experience, as stated by the best practical writers on the dietetics of children. They have all told us that pure and fresh milk from the cow is the best substitute for that of the mother. But I am fully satisfied, both from analytical investigations and clinical observation, that most of these writers have committed one important error; which consists in recommending the milk of the cow to be used largely diluted with water, and sweetened by the addition of sugar. Thus Dr. Dewees recommends it, for infants under five months, to be diluted with one part of water to two parts of milk and the addition of a little sugar. To children over five months he allows the use of rice and gum water in addition to the diluted milk. Dr. Condie in his work on Diseases of Children, says: "The quantity of (cow's) milk required for use should be mixed with nearly an *equal quantity* of warm water and *well* sweetened with sugar." Drs. Churchill, Pareira, and others coincide in nearly the same recommendation.

The foundation for this recommendation is the supposed fact that cow's milk contains more butter and caseine and much less sugar than woman's milk. This impression was made on the older writers, by observing on woman's milk when allowed to stand, the appearance of a smaller quantity of cream and curd than on cow's milk. The same has been perpetuated by the analysis of woman's milk published by M. M. Chevallier and Henri in 1839, and quoted as the standard of comparison by Pareira, Churchill, and others. The result of their analysis is stated as follows:

1000 parts of woman's milk gave of water 879,8; solid constituents 120,2. The latter consists of

| | |
|---|---|
| Butter,............... | 35,5 |
| Sugar of milk,......... | 65,0 |
| Caseine,.............. | 15,2 |
| Salts,................ | 4,5 |

Satisfied from my own analyses that this statement does not correctly represent the average relative proportion of caseine and sugar in healthy human milk, I extended the inquiry and found that it differed equally from the results of analyses by Simon, Clemm, and still more recently by M. M. Vernois and Becqueral. Simon made fourteen analyses of woman's milk at different periods of gestation, and with the following average result: In 1000 parts, Water, 883,6; Solid matter, 116,4.

The solid matter contained, of

| | |
|---|---|
| Butter,............. | 25,3 |
| Sugar and Ext.,..... | 48,2 |
| Caseine,............ | 34,3 |
| Salts,.............. | 2,3 |

Clemm made three analyses during the first two weeks of lactation, and gives us the relative proportion in 1000 parts of sugar and extractive matter, 41,1; of caseine, 35,3.

M. M. Vernois and Becqueral, state the relative proportion of the same ingredients to be, of sugar 43,6, and caseine 39,2.

It will be seen, therefore, that the results obtained by all these differ but little from those obtained by myself as stated in the first part of this report. If we compare the results obtained by me with those just given, we may deduce the following table, which will probably afford as reliable a representation of the average composition of healthy human milk as can be obtained:

| | |
|---|---|
| Water,................. | 885,50 |
| Solid matter,........... | 114,50 |
| Of which there is | |
| Butter,................. | 29,70 |
| Sugar and Extractive,.... | 43,35 |
| Caseine,................ | 38,27 |
| Salts,.................. | 7,20 |

This compared with the table given by Chevallier and Henri, as quoted so generally by writers on dietetics, represents the relative proportion of sugar as one-third less and that of caseine as more than double; and if compared with cow's milk in reference to the three important classes of alimentary principles would exhibit the following result, viz:

| | Nitrogenous Elements. | Carbonaceous. | Saline. | Water. |
|---|---|---|---|---|
| Woman's milk,........... | 38,27 | 73,05 | 2,30 | 885,50 |
| Cow's milk,.............. | 44,80 | 79,00 | 6,00 | 870,09 |

It will thus be seen that the composition of cow's milk, whether viewed in relation to the absolute amount of solid constituents, or to the relative proportion of its several proximate elements, affords no indication for more than a slight dilution with water accompanied by a correspondingly small addition of sugar. During the progress of my investigations concerning the properties of milk, two facts were observed which led to farther inquiries. The first was, that fresh human milk gave to litmus a much more decided alkaline re-action than cow's milk; and when allowed to remain in an open vessel, remained entirely fluid and without any indications of sourness for a much longer time. The second was, that in almost all instances in which infants were made to depend on cow's milk for nourishment and they did not thrive well, the failure seemed to be accompanied by a speedy and excessive generation of acid in the digestive apparatus, indicated by either vomiting of some milk soon after it was taken, or too frequent stools of a curdled appearance and sour smell. These facts led to the inquiry whether a large part of the difficulty arising from the use of cow's milk for infants, did not depend directly on the more ready coagulability of its caseine, as compared with the milk of the mother.

To test the relative coagulability and fermentive tendency of the two kinds of milk, I placed a specimen of woman's milk and of cow's milk in vessels of equal size and shape side by side, and allowed them to remain at rest three days. The specimen of cow's milk emitted an acid smell and re-action, together with a visible coagulation of the caseine in 36 hours, while the specimen of woman's milk remained apparently perfectly sweet and free from coagulation at the end of 48 hours.

To determine the effect produced on cow's milk by aqueous dilution, I placed side by side in equal quantities and equal vessels, three specimens; one of pure cow's milk, another of milk of the same cow diluted with half its quantity of water, and a third diluted with the same quantity of water and the addition of a little white sugar. Of these specimens, that which was simply diluted with water showed signs of fermentation and coagulation first, next that diluted with water with the addition of sugar, and last the pure milk. Having satisfied myself by these and other experiments, that the caseine of the milk of the cow is held in solution by a feebler affinity than that of the human female; and that simple dilution with water, or water and sugar, only renders this affinity still more feeble, and thereby favors both fermentation and coagulation; my next inquiry was, whether any substances could be added to the milk of the cow which would positively strengthen the affinity by which the caseine is held in solution and thereby materially retard both coagulation and fermentation. It being supposed that caseine in fresh milk is kept soluble by union with a certain proportion of soda, and that while fresh it always gives an alkaline reaction though feebler than that given by woman's milk, we should be disposed to look first to the class of alkaline substances as most likely to produce the desired effect. Accordingly four specimens of fresh cow's milk were placed in vessels of equal size and shape. To one was added chloride of sodium (common salt) in the proportion of 5 grs. to the oz. of milk; to another an equal proportion of bi-carbonate soda; to a third, aqua calcis (lime water) in the proportion of half-a-fluid drachm to the oz. of milk; while the fourth specimen remained without the addition of anything. After standing 36 hours the 4th specimen emitted a distinct sour smell and showed a visible coagulation of the caseine. The third specimen did not exhibit the same changes until the end of 53 hours; while the 1st and 2nd specimens remained without any perceptible change full 72 hours. These results were striking and susceptible of a highly important practical application. They demonstrated the practicability of keeping the caseine of cow's milk soluble a much longer time by the addition of alkalies, and thereby rendering it more nearly identical in its properties with the milk of the human female. From all the foregoing observations and experiments, we are led to the following inferences, viz:

1st. That cow's milk more nearly approximates, in composition and properties, the milk of the human female than any other substance; and is consequently better adapted to the feeding of infants than any other substances in use.

2nd. That in preparing cow's milk for the feeding of infants less than six months old, it shonld be diluted with not more than *one-fourth* part of water, and receive the addition of from three to five grains of common salt or bi-carbonate of soda to the ounce of milk, and sufficient sugar to give it a slightly sweetish taste. For infants over six months old, the proportion of water should not be more than one-sixth.

During the last three or four years I have regulated the food of infants coming under my care, in accordance with the foregoing propositions, and with the most satisfactory results. The solidified milk, manufactured in Dutchess County, New York, contains just about the required amount of additional soda and sugar, and when dissolved in the right proportion of water, makes the best food that I have yet used for infants deprived of the mother's milk. I have now, within my circle of patients, three infants growing finely on the solidified milk exclusively, and have been since they were from three to six weeks old. It not only contains the needed addition of soda and sugar, but being perfectly dry, and capable of being kept any length of time, it can always be dissolved fresh for use whether in winter or summer; and consequently affords greater uniformity than can be procured by any other article in use. I have met with two or three children, who would not thrive well on any preparation of milk that I could devise.

With these, the oatmeal or groats, answered a better purpose than any other kind of food.

Dr. W. H. Cumming, who has recently published an interesting work on the dietetics of children, gives the following directions for preparing cow's milk for infants:

"Take, then, ordinary cow's milk and let it stand for four or five hours. For a child three months old, 2¼ quarts will be needed. Take the upper third, (3½ pints,) and add to it 2¼ pints of water; sweeten it with the best sugar, of which 2⅝ ounces will be required. It should be made somewhat sweeter to the taste than ordinary cow's milk.

A child three months old will take from 48 to 60 fluid ounces, daily, in six or seven doses of a half pint each.

It should be given from a bottle—*suction being the only proper mode of feeding for a young child.*

Its temperature should be from 100° to 104°. It should be warmed again if it becomes cool while the child is taking it.

The child should be early trained to pass 6 or 8 hours at night without feeding.

The kind of bottle, which for cheapness and convenience is most advantageous, is a plain 8 ounce vial, of an elliptical form. The artificial nipple is made best by rolling a quill in soft muslin and forcing this into the neck of the vial, leaving about three-fourths of an inch projecting from the neck. The ease with which the muslin may be unrolled and thoroughly washed, gives this arrangement a superiority over every other, especially in warm weather. The quill also may be readily cleaned.

The child should be fed at intervals of three or three and a half hours. Regularity in this respect is very advantageous.

During the first month, the child needs food of different composition. There should be more butter in proportion to the caseine. In order to obtain this increased proportion of butter, let the upper *eighth* of the milk be taken instead of the upper *third.* This milk contains from 70 to 80 thousandths of butter. It should be diluted with 2.6 parts of water."

| | | | | | | | |
|---|---|---|---|---|---|---|---|
| For a child from | 3 to 10 days old. | Mix | 1000 | Water | 2643 | Sugar | 243 |
| " " | 10 to 30 " | | " | " | 2500 | " | 225 |
| " " | 1 month old. | | " | " | 2250 | " | 204 |
| " " | 2 " | | " | " | 1850 | " | 172 |
| " " | 3 " | | " | " | 1500 | " | 144 |
| " " | 4 " | | " | " | 1250 | " | 124 |
| " " | 5 " | | " | " | 1000 | " | 104 |
| " " | 6 " | | " | " | 875 | " | 94 |
| " " | 7 " | | " | " | 750 | " | 84 |
| " " | 9 " | | " | " | 675 | " | 78 |
| " " | 11 " | | " | " | 625 | " | 73 |
| " " | 14 " | | " | " | 550 | " | 67 |
| " " | 18 " | | " | " | 500 | " | 63 |

By thus gradually diminishing the proportion of water, we furnish the child a milk containing an ever-increasing proportion of nutritive matter.

This method of Dr. Cummings, is founded on the supposition that it is desirable to increase the relative proportion of butter in the milk of the cow, and at the same time greatly diminish that of the caseine. Without stopping to inquire whether this supposition is well founded or not, it is sufficient to know that the method is impracticable in those localities and among those classes where artificial food for infants, is most needed, namely, in all our large cities. Nine-tenths of the inhabitants of all our cities, obtain their milk from the regular *milk-men*, whose supply consists of the milk of many cows mixed together; first shook up over many miles of railroad; then diluted more or less with water and ice, and shook up some hours during the delivery along the paved streets of a populous town. To talk of letting such milk stand a certain length of time in order to obtain the "*upper-third*," for the purpose of feeding an infant, is preposterous; at least, during the summer months. In spite of *scalding* and refrigerators both, every family who has tried it, knows that it is difficult to keep such milk sweet from morning until evening, and that it is often actually sour in less than an hour after delivery.

From close observation through a series of years I am satisfied that the excessive dilution of milk, and the use of rice-water, barley-water, bread-water, and such other preparations as require the child to take a large quantity of water to get a small amount of really nutritious matter, greatly favors the prevalence of cholera-infantum and diarrhœa, during all the warm season of the year. Indeed, the excessive use of liquids, is a prominent cause of intestinal fluxes, in the old as well as the young.

The heat of the summer relaxes all the tissues more or less, and renders the cutaneous and mucous surfaces more sensitive than normal. If at the same time water or diluted drinks are freely taken, the skin and kidneys secrete more largely; not only discharging the excess of water from the blood, but also a considerable quantity of saline matter with it. The supply of water being kept up, it is easy to see, that the saline matter of the blood would soon become deficient, and consequently the capacity of that fluid for the absorption of oxygen from the air-cells of the lungs, would be impaired. With a deficiency of both salts and oxygen in the blood, with an excess of water, and a relaxed and sensitive state of the mucous surfaces, we have a state of things highly favorable to the occurrence, of cholera-morbus, diarrhœa, and dysentery. So fully am I satisfied of the correctness of these views, that I do not hesitate to call the attention of the profession to them, especially in regard to their bearing on the preparation of food for infants.

